# Effects of pretreatment with methanol extract of Peucedani Radix on transient ischemic brain injury in mice

**DOI:** 10.1186/s13020-017-0151-z

**Published:** 2017-10-24

**Authors:** So-Youn Jung, Kyoung-Min Kim, Suin Cho, Sehyun Lim, Chiyeon Lim, Young Kyun Kim

**Affiliations:** 1 College of Korean Medicine, Dong-Eui University, Yangjeong-ro, Busanjin-gu, Busan, 47227 Republic of Korea; 2 School of Korean Medicine, Pusan National University, Yangsan, Gyeongnam 50612 Republic of Korea; 3 School of Public Health, Far East University, Chungbuk, 27601 Republic of Korea; 4 College of Medicine, Dongguk University, Ilsandong-gu, Gyeonggi-do 10326 Republic of Korea

**Keywords:** Peucedani Radix, *Angelica decursiva*, Stroke, Anti-inflammation

## Abstract

**Background:**

Stroke is the second most common cause of death and may result in various disabilities; thus, identification of neuroprotective therapeutic agents is important. Peucedani Radix (PR), the root of *Angelica decursiva*, is a well-known remedy for damp and phlegm in Korean medicine and has also been shown to exert antioxidant and anti-inflammatory activities. This study was performed to investigate the mechanism underlying the anti-inflammatory effect of methanol extract of PR (PRex) on cerebral ischemic injury.

**Methods:**

C57BL/6 male mice were orally administered PRex (20, 60, or 200 mg/kg) at 2 days, 1 day, and 1 h prior to middle cerebral artery occlusion (MCAO). Twenty-four hours after MCAO, the infarct volume was measured and the neurological deficit score was assessed. The inflammatory-related substances in the ipsilateral hemisphere were determined by western blotting, DCFH-DA assay, TBARS assay, and ELISA.

**Results:**

PRex pretreatment significantly decreased the infarct volume at 24 h after MCAO. Moreover, PRex effectively suppressed the expression of iNOS, ROS, MDA, and pro-inflammatory cytokines, such as IL-1β and TNF-α, in brain tissue of mice with MCAO-induced brain injury.

**Conclusions:**

PRex protected neurons from ischemic brain injury in mice through its antioxidant and anti-inflammatory activities. Our results suggested that PR could be a promising candidate in the therapy of ischemia-induced brain damage.

**Electronic supplementary material:**

The online version of this article (doi:10.1186/s13020-017-0151-z) contains supplementary material, which is available to authorized users.

## Background

The clinical signs of stroke, which arise from a focal disorder of cerebral function that results from the occlusion of blood vessels or hemorrhage, include diverse speech and motor disorders or death [1, 2]. As the quality of life for patients and their families may be irreversibly diminished, the prevention and treatment of stroke are very important, and results from stroke are highly correlated with the extent of brain tissue damage from oxidative damage and neuroinflammation [3, 4]; thus, research into anti-inflammatory and neuroprotective therapeutic interventions is warranted.

To investigate the efficacy of therapeutic agents against ischemia-induced brain damage, which accounts for approximately 80% of the types of stroke [5], an animal model with similar pathological changes as those in the human body is necessary. The rodent model of middle cerebral artery occlusion (MCAO), which is reproducible and minimally invasive, is commonly used in stroke research [6, 7]. The intraluminal filament method devised by Koizumi et al. [8], and later modified by Longa et al. [9], has been widely used because it allows reperfusion post occlusion [10]. Gupta et al. studied the neuroprotective effect of a combination of *Zizyphus jujuba* and silymarin [11], Gim et al. researched the antioxidant activities of curcumin treatment in rats with MCAO-induced cerebral ischemia [12], and Na et al. reported the antioxidant and anti-inflammatory activities of 6-shogaol pretreatment in MCAO-injured mice [13].

Peucedani Radix (PR), the root of *Angelica decursiva* Franchet et Savatier, has been used traditionally as a remedy in Korean medicine for thick phlegm, cough, asthma, and upper respiratory tract infections [14]. Many studies of the pharmacological activities of PR have been recently undertaken. Lim et al. found that the methanol extract of PR had significant inhibitory activity against lung inflammation [15] and Zhao et al. reported that the constituents of PR, predominantly umbelliferone 6-carboxylic acid, could be used for the treatment of oxidative stress-related inflammatory diseases [16]. Coumarins from *A. decursiva* were demonstrated to be effective in the treatment of type 2 diabetes and inflammation-associated disorders [17, 18]. These studies demonstrated the antioxidant and anti-inflammatory activities of PR, which may offer a potential treatment for cerebral damage as oxidative stress and inflammation are responsible for ischemic injury and can eventually result in neuronal death [19]. Although the various cytoprotective actions of PR have been extensively investigated, its effects against ischemia-induced brain damage have not been determined. Hence, to determine whether PR inhibits ischemic brain damage, we observed infarct volumes, neurological deficits, and inflammatory mediators in mice with MCAO-induced brain injury after PRex pretreatment.

## Methods

### PR treatment

PR was purchased from Naemomedah (Kwangmyoungdang Medicinal Herbs, Ulsan, Korea) and authenticated by Dr. Cho (Pusan National University School of Korean Medicine, Yangsan, Korea). Due to difficulties of maintaining consistent qualities of herbal extracts, fingerprinting data of the PRex were obtained for future study using high performance thin layer chromatography (HPTLC) method (Additional file 1: Figure S1). A voucher specimen (No. PD16-0322) was deposited in the low temperature room of the laboratory. For solvent extraction, PR (200 g) was immersed in methanol (1000 mL), left at room temperature for 5 days, and the supernatant fluid was collected. This process was repeated for the PR residue. The first and second supernatant fluid were filtered with filter paper and concentrated to dryness; finally, a total of 64 g PR extract (PRex) was obtained (32% yield). PRex was dissolved in dimethyl sulfoxide (DMSO), diluted with 0.9% normal saline, filtered through a 0.45-μm pore sized syringe filter, and adjusted to the concentrations of 20, 60, and 200 mg/kg.

### Animal model

**Fig. 1:**
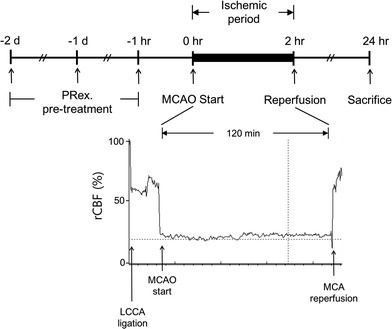
Design of the MCAO model. The mice were pretreated with methanol extract of Peucedani Radix (PRex) for three consecutive days before MCAO and the mice were killed 24 h after MCAO. During the ischemic conditioning, the relative cerebral blood flow (rCBF) was monitored by using Laser-Doppler flowmetry. *LCCA* left common carotid artery, *MCA* middle cerebral artery

The experimental protocol involving animals was approved by the ethics committee of PNU (Pusan National University; Approval Number PNU-2016-1087). The Minimum Standards of Reporting Checklist (Additional file 2) contain details of the experimental design, and statistics, and resources used in this study. Male SPF C57BL/6 mice (Daehan Biolink, Chungbuk, Korea) (22–25 g) were housed in a temperature- and humidity-controlled environment under a 12-h light/dark cycle and given food and water ad libitum for at least 7 days prior to the experiment. Three mice were housed in each cage. The mice were randomly divided into five groups with a minimum of eight mice in each group: the sham control group, in which the animals underwent surgery, but were not subjected to MCAO; the MCAO control group, in which the animals did not receive PRex pretreatment, but were subjected to MCAO; and the 20, 60, and 200 mg/kg PRex pretreated MCAO groups, in which the animals received PRex treatment at 20, 60, or 200 mg/kg, respectively, and were subjected to MCAO. The animals were orally administered 20, 60, or 200 mg/kg of body weight at 2 days, 1 day, and 2 h prior to the MCAO procedure (Fig. 1); in the other groups, mice received an equivalent amount of normal saline instead of PRex. Mice in the MCAO control group and the PRex groups were subjected to MCAO. Isofluorane gas (2%) was added to a mixture of 70% N_2_O and 30% O_2_ to produce the inhalation anesthetic. During the operation, rectal temperature was maintained at 36.5 ± 0.5 °C via a heating pad and relative cerebral blood flow (rCBF) was monitored using a Laser-Doppler blood flow system (moorVMS-LDF, Moor Instruments, Devon, UK). The general procedure of Koizumi et al. [8] was employed, with some modifications. The hair on the chest and neck of the animal was cleanly removed using a clipper and the skin was cut. After the branches of the left common carotid artery (LCCA) were confirmed, the left external carotid artery (LECA), left internal carotid artery (LICA), and surrounding connective tissues were carefully arranged under a stereo microscope (Nikon 745, Tokyo, Japan) to secure a clear view.

The LECA and the LCCA were bound with 4/0 silk sutures (Ethicon Inc., NJ, USA) to temporarily obstruct the flow of the LICA. A filament (11-mm length of 8/0 nylon suture with a silicon-coated tip, Ethicon, Scotland) was inserted slowly through the LICA to the origin of the LMCA to occlude the LMCA. The inserted filament was fixed with blood vessels and left for 2 h to ensure an ischemic period. Subsequently, the filament was removed to allow reperfusion. During the 2-h period, rCBF was reduced to < 20%, but sharply increased to > 90% of baseline during reperfusion, which indicated that the MCAO procedure was successful. The skin was immediately sutured and the animal was awakened from anesthesia. In the sham control group, the sham operation consisted of binding of the LECA and ligation of the LCCA, but the LMCA was not occluded; finally, the incised muscle and skin were sutured. The mice were euthanized by CO_2_ inhalation at 24 h after MCAO.

### Measurement of infarct volume

The brains harvested at 24 h after the MCAO procedure were immediately sliced into 1-mm coronal sections from the olfactory bulb to just before the cerebellum and 10 sections were cut per brain. The sections were incubated with 2% 2,3,5-triphenyltetrazolium chloride (TTC) solution for 17 min at 25 °C and then soaked in 10% neutral buffered formalin for more than 2 h. Relative edema and infarct volumes were computed by Image J software (NIH, Maryland, USA) from digital images obtained by a digital camera.

### Neurological deficit scores

Twenty hours after MCAO, neurological deficit scores were assessed by using the following five-point scale: 0, no neurological deficit; 1, an incomplete extension of right forepaw and a reduced grip when tail pulled; 2, voluntary movement in all directions and turning to the right when tail pulled; 3, walking or circling to the right and sensitive to nociception when stimulated; 4, no response to stimulation or stroke-related death.

### Western blot analysis

The mice brains of ischemic hemisphere were dissected and homogenized in modified phosphate buffered saline (PBS) containing 150 mM NaCl, 1 mM EDTA, 50 mM Tris, and 1:100 (v/v) of proteinase inhibitor. The expression level of inducible nitric oxide synthase (iNOS) in the mouse brains was then assessed by western blotting.

Total proteins were isolated using a protein extraction solution (pro-prep, iNtRON, Gyeonggi-do, Korea). The cell lysates were obtained by centrifugation at 13,250×*g* for 10 min at 4 °C. Equal amounts of proteins were separated in sodium-dodecyl sulfate polyacrylamide gels and transferred to PVDF membranes (Millipore, Darmstadt, Germany), which were blocked using 5% skim milk in TBST buffer for 1 h at room temperature and then incubated overnight at 4 °C with specific antibodies for iNOS (1:500) and β-actin (1:1000). Subsequently, the membranes were incubated with horse radish peroxidase (HRP)-conjugated goat anti-rabbit IgG pAb (1:5000) and HRP-conjugated goat anti-mouse IgG pAb (1:3000) for 2 h. The membranes were then treated with ECL solution (GenDEPOT, Houston, TX, USA) and the protein bands were detected by a photosensitive luminescent analyzer system (Amersham™ Imager 600, UK). The band intensities were analyzed using Image J (NIH, Maryland, USA) to determine the relative protein quantities in comparison with β-actin. iNOS antibody were obtained from Cell Signaling (Danvers, MA, USA), and secondary antibodies goat anti-rabbit IgG pAb was obtained from Enzo Life Sciences (Farmingdale, NY, USA).

### Inflammatory cytokine analysis

The brain tissue of ischemic hemisphere was homogenized in PBS (pH 7.4) (5% w/v) and the resultant homogenates were clarified at 10,000×*g* for 5 min at 4 °C. The post-mitochondrial supernatants were obtained by a second centrifugation step at 10,000×*g* for 20 min at 4 °C and used for enzyme-linked immunosorbent assays (ELISAs) (Abcam, Cambridge, MA, USA). The levels of IL-1β and TNF-α in the brain tissue were measured by ELISA using a commercially available kit in accordance with the manufacturer’s instructions. The detection limit of the assay was 0.1 ng/mL. The absorbance of the reaction products at 450 nm was measured by using a microplate reader.

### Determination of ROS

To measure the production of reactive oxygen species (ROS) and malondialdehyde (MDA), ROS generation in the brain was determined in tissue homogenates by using dichlorofluorescein diacetate (DCFH-DA) [20]. The tissue homogenate was incubated with 1 mM 2′,7′-dichlorodihydrofluorescein diacetate for 30 min at 37 °C. The absorbance was measured by using a fluorescence microplate reader at an excitation wavelength of 485 nm and an emission wavelength of 535 nm.

### Estimation of oxidative stress markers

To measure oxidative stress in the injured brain tissue, level of malondialdehyde (MDA), a biomarker of lipid peroxidation, was estimated. The level of MDA in the injured hemispheres was examined using the TBARS (thiobarbituric acid-reactive substances) Assay Kit (Cayman Chemical, Ann Arbor, MI) [21]. The optical density (OD) was read at 540 nm by a spectrophotometer and the results were defined as μM/μg wet tissue.

### Histological staining

The harvested brain tissue was fixed in 10% formalin, dehydrated by alcohol, and embedded in paraffin. The sections of the tissue were cut into 3-μm thick slices on the glass slides, deparaffinized by xylene, and stained with hematoxylin and eosin (H&E) or cresyl violet (CV) to observe the histological changes in brain tissue after MCAO-injury by using a microscope (ZEISS AXIO, Carl Zeiss, Oberkochen, Germany).

### Statistical analysis

One-way ANOVA was used to determine the statistical significance of differences. The data were expressed as the mean ± standard deviations (STDEVs). SPSS 23.0 version was used to perform the statistical analyses and p values of ≤ 0.05 were considered statistically significant.

## Results

### Effects of PRex on infarct volumes and behavioral deficits

**Fig. 2:**
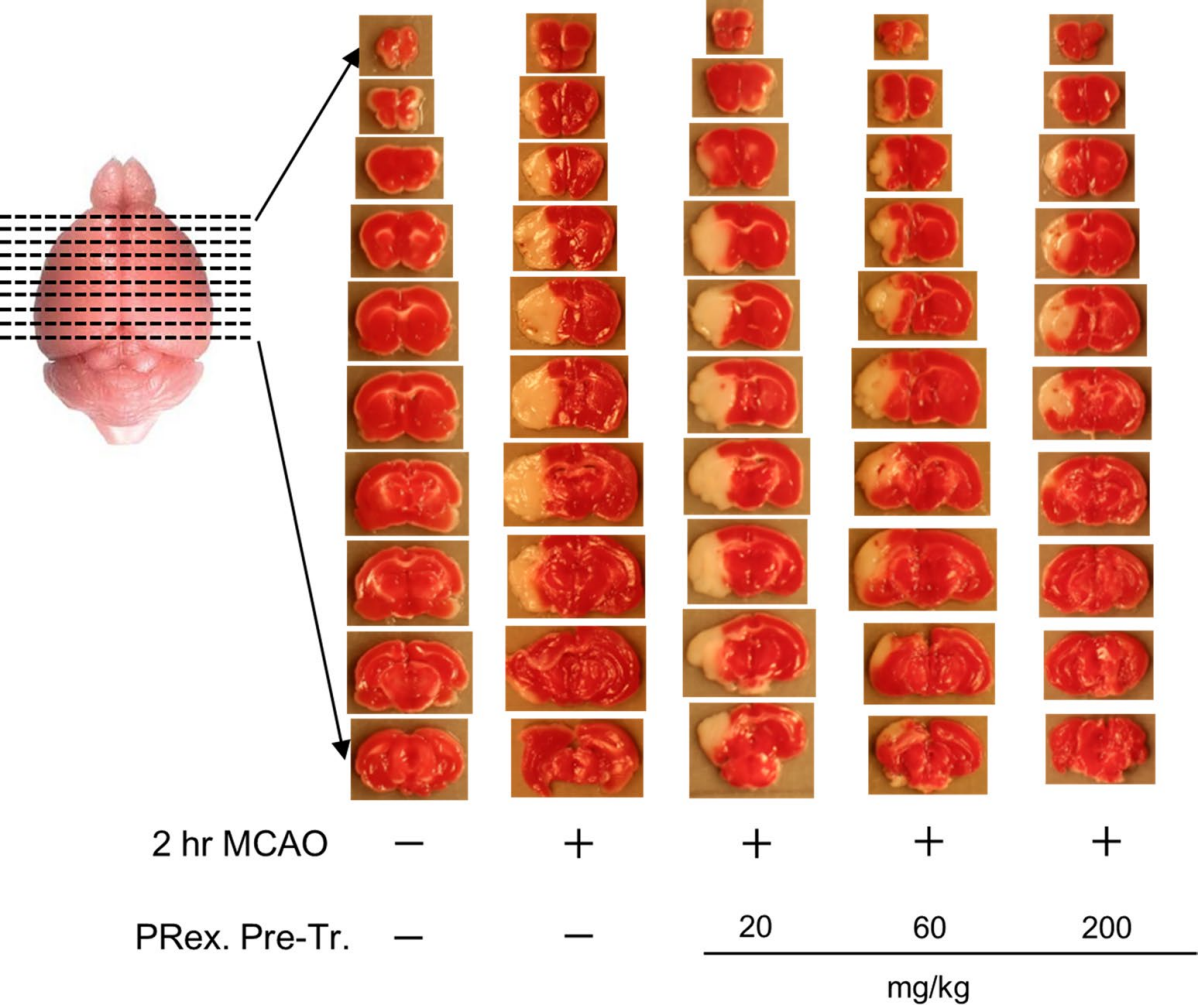
Representative group images of ischemic lesion volume detected by TTC at 2 h after MCAO-induced brain injury. The harvested brain slices were stained with TTC to measure the infarct volume. The ischemic regions were identified as pale regions in the coronal slices. TTC, 2,3,5-triphenyltetrazolium chloride

**Fig. 3:**
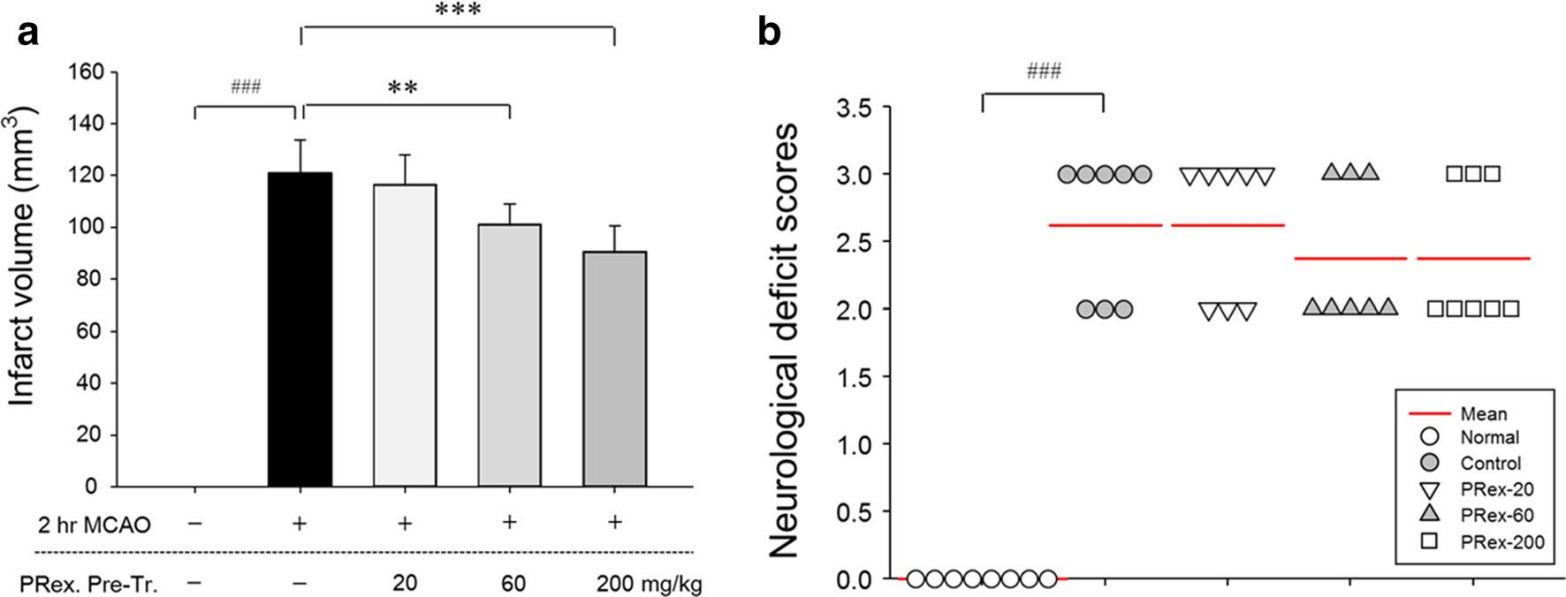
Effects of PRex pretreatment on infarct volumes (**a**) and neuronal deficit score (**b**) in the brains of MCAO-injured mice. The PRex pretreatment significantly decreased the infarct volumes at 24 h after MCAO. But pretreatment of PRex did not improve neuronal deficit scores. The results are presented as the mean ± SD. ^###^p < 0.001 vs sham control group, **p < 0.01, ***p < 0.001 vs MCAO control group; n = 8 in each group

The regions of infarction were represented by TTC staining. The sham operation induced no damage, but MCAO caused a relatively wide range of damage in the ipsilateral hemispheres (mean ± SD; 121.167 ± 12.671 mm^3^). However, significantly smaller infarct lesions were found in the 60 or 200 mg/kg PRex pretreated MCAO groups than in the MCAO control group (60 mg/kg, 101.0 ± 7.874 mm^3^; 200 mg/kg, 97.167 ± 9.867 mm^3^) (Figs. 2, 3a). The neuronal deficit scores were significantly higher in mice with MCAO. PRex pretreatment did not significantly reduce motor behavioral scores compared with the MCAO control (Fig. 3b).

### Effects of PRex on iNOS expression

**Fig. 4:**
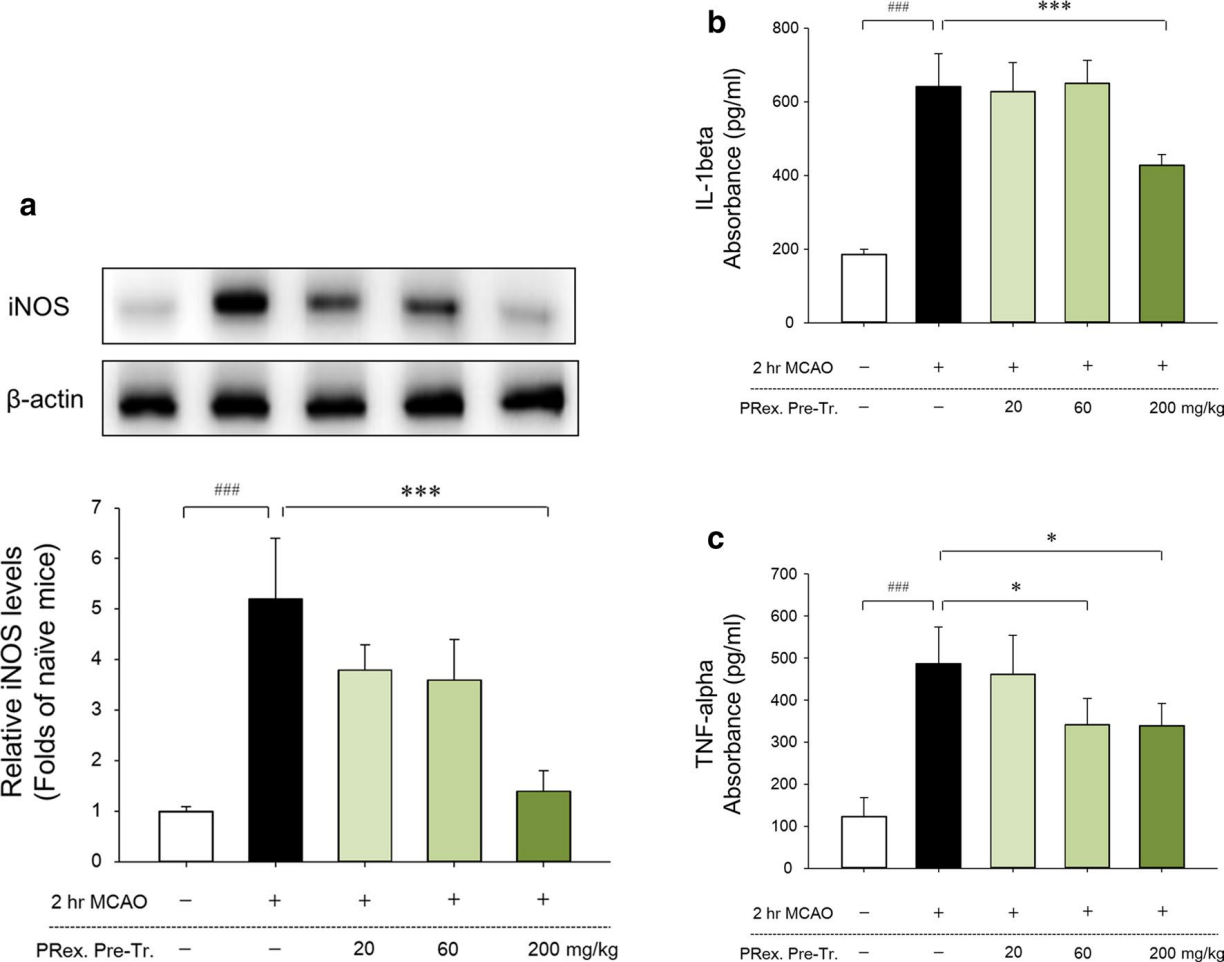
Effects of PRex pretreatment on iNOS (**a**), IL-1β (**b**), and TNF-α (**c**) in the brains of MCAO-injured mice. PRex pretreatment significantly decreased iNOS level in the mouse model of ischemic brain stroke (**a**). Representative western blots and quantitative analysis of iNOS expression show the effect of PRex on iNOS expression in brain tissue. The IL-1β and TNF-α levels were measured by using a commercially available ELISA kit. The results are presented as the mean ± SD. ^###^p < 0.001 vs sham control group, *p < 0.05, ***p < 0.001 vs MCAO control group; n = 8 in each group

As determined by the western blot analysis to identify protein changes in harvested brain sections at 24 h after MCAO, the inducible NOS (iNOS) level was significantly higher than that of the sham controls. However, mice in the 200 mg/kg group had significantly lower iNOS levels than did the mice in the MCAO control group (Fig. 4a).

### Effects of PRex on pro-inflammatory cytokine levels

The effects of PRex on brain inflammation after MCAO injury were evaluated by the measurement of the levels of pro-inflammatory cytokines. The brains of MCAO-injured mice exhibited higher concentrations of IL-1β (mean ± SD: 642 ± 89 pg/mL) and TNF-α (487 ± 87 pg/mL) compared with levels in the sham-operated mice (IL-1β, 186 ± 13 pg/mL; TNF-α, 123 ± 45 pg/mL). However, PRex pretreatment significantly suppressed the expression of IL-1β (200 mg/kg, 428 ± 29 pg/mL) and TNF-α (60 mg/kg, 342 ± 62 pg/mL; 200 mg/kg, 339 ± 53 pg/mL) (Fig. 4b, c).

### Effects of PRex on ROS production and lipid peroxidation

**Fig. 5:**
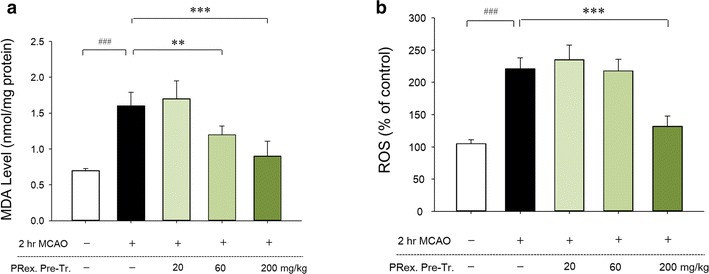
Effects of PRex on ROS (**a**) and MDA levels (**b**) in the brains of MCAO-induced mice. The results are presented as the mean ± SD. ^###^p < 0.001 vs sham control group, **p < 0.01, ***p < 0.001 vs MCAO control group; n = 8 in each group

To examine the antioxidant effect of PRex, the contents of ROS and MDA in the brain were measured. The ROS and MDA levels were significantly elevated in the MCAO group (mean ± SD: ROS, 221 ± 17%; MDA, 1.60 ± 0.19 nmol/mg) compared with that in the sham operated group (ROS, 105 ± 6%; MDA, 0.70 ± 0.03 nmol/mg). However, the ROS (200 mg/kg, 132 ± 16%) and MDA levels (60 mg/kg, 1.20 ± 0.12 nmol/mg; 200 mg/kg, 0.90 ± 0.21 nmol/mg) were significantly lower in the PRex-administered groups (Fig. 5a, b).

### Effects of PRex on histological changes in brain tissue

**Fig. 6:**
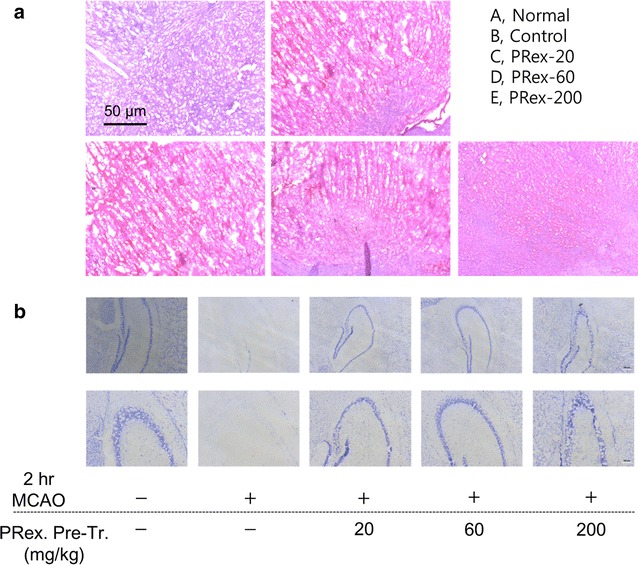
Effects of PRex pretreatment on histological change in the brains of MCAO-injured mice stained by hematoxylin and eosin (**a**), and by cresyl violet (**b**). Red staining indicates nuclear damage. Neurons dyed by cresyl violet are stained purple

To confirm the histological changes in brain tissue 24 h after MCAO, the tissue was stained by H&E or CV. The brain tissue was more strongly stained red by H&E in the MCAO control group than in the sham controls. At the highest concentration of PRex, the brain tissue was more strongly stained blue than that observed for the MCAO control group (Fig. 6a). In the MCAO control group, fewer purple-stained neurons were observed than in the sham controls; however, in the PRex pretreatment groups, they appeared more often than in the MCAO control group (Fig. 6b).

## Discussion

Globally, stroke has been the second most common cause of death for 15 years; more than six million deaths were attributed to stroke in 2015 [22]. Ischemia-induced brain damage accounts for approximately 80% of the types of stroke^5^ and may result in permanent disability, which severely impairs dependence and precipitates a large social expense. Furthermore, the incidence of stroke in young adults is experiencing an upward trend [23]. Therefore, the prevention and recovery of stroke are important issues.

In Korean medicine, ischemic brain damage is one of the diseases included in sudden hit by the wind (卒中風) and classified as fire-heat pattern (火熱證), Yin deficiency pattern (陰虛證), Qi deficiency pattern (氣虛證), and the dampness-phlegm pattern (濕痰證) [24]. The dampness-phlegm pattern of stroke has a significant relationship with metabolic syndrome and obesity [25], which are prominent causes of stroke.

PR is a representative herb that expels damp and phlegm, and it has long been used as a therapeutic agent for thick phlegm, upper respiratory tract infections, and asthma [14]. PR was not mentioned as a major therapeutic agent, but it has been reported to exert antioxidant and anti-inflammatory activities [15–18, 26], which are potentially related to the treatment of ischemia-induced brain damage.

When stroke occurs, the cell death that results from the deprivation of oxygen and glucose consequently results in cerebral damage [27, 28]. Although various mechanisms participate in the pathological process of stroke, considerable evidence has indicated that inflammation plays an important role in its progression [29–31]. The inflammatory responses caused by excitotoxicity and oxidative stress owing to hypoperfusion in ischemic areas lead to blood–brain barrier dysfunction and cell death [32].

Although several pharmacological agents were found to be effective in animal models, they did not work in humans [33, 34]. Laboratory experimental conditions did not reflect the factors that influenced stroke in the human population, nevertheless, the most applicable and frequently chosen animal models for research stroke are rodents [33, 35]. The MCAO injury to rodents, the most commonly used surgical procedure to produce stroke, damages the subcortical and cortical structures that mimic human cerebral infarcts in terms of the size and the affected structures [36]. Inflammation has been reported to play an important role in the pathogenesis of ischemic stroke; the brain responds to ischemic injury with an acute and prolonged inflammatory process that can be characterized by the rapid activation of resident cells, the production of pro-inflammatory mediators, and the infiltration of various types of inflammatory cells [37]. Over 1000 drugs have been tested in animal models; of these, 114 underwent clinical evaluation, but a larger proportion of the agents studied previously have failed. Despite the many clinical trials conducted, rt-PA remains the only agent shown to improve stroke outcome and therefore the optimum treatment of cerebral focal ischemia has remained as one of the major challenges of clinical medicine [33–38].

In this study, we evaluated the anti-inflammatory effect of PRex on ischemia-induced brain damage in a mice model. PRex pretreatment was found to reduce the infarct volumes in mice brains 24 h after MCAO (Figs. 2, 3a), but did not significantly improve the neurological behavioral deficits (Fig. 3b). In addition, histological staining indicated that PRex pretreatment protected the nucleus and neuronal cells. Hematoxylin stains the nucleus blue and eosin stains the cytoplasm red; hence, tissues with damaged nuclei appear red. CV stains neurons; in this study, the lighter purple staining indicates greater damage to the neurons of the brain tissues around the hippocampus. In the present study, the tissue stained red by H&E after MCAO injury became more blue as the concentration of PRex pretreatment increased (Fig. 6a) and the reduction in the area stained purple by CV after MCAO-induced brain injury was increased in the PRex pretreatment groups (Fig. 6b).

Nitric oxide synthase (NOS) enzymes, including endothelial NOS (eNOS), neuronal NOS (nNOS), and inducible NOS (iNOS), are important in the maintenance of homeostasis through the production of NO from l-arginine. eNOS and nNOS produce low physiological levels of NO; iNOS consistently produces large amounts of NO through inflammatory cytokines and bacterial products. NO can exert cytotoxicity through the formation of strong oxidant peroxynitrite with superoxide, especially in inflammatory responses. iNOS promotes inflammation and acts synergistically with other inflammatory mediators. In the brain, the increase in iNOS mRNA and protein after ischemia led to NO production and DNA damage. Therefore, the inhibition of iNOS activity could be critical for the suppression of inflammation in the ischemic brain [39–44]. In the current study, PRex pretreatment was shown to reduce the effects on increased iNOS expression in MCAO-induced brain damage (Fig. 4a).

Among the cytokines known to be associated with inflammation in stroke [45], interleukin-1 (IL-1) and TNF-α were reported to aggravate cerebral injury; in contrast, interleukin-6 (IL-6), interleukin-10 (IL-10), and transforming growth factor-β (TGF-β) were shown to be neuroprotective [46]. In the early stage of focal cerebral ischemia, pro-inflammatory cytokines, such as IL-1β and TNF-α, promote the expression of adhesion-like glycoprotein P and E-selectin and the stimulation of leukocytes attached to the activated vascular endothelium [47]. In the present study, PRex was found to significantly ameliorate the MCAO-induced upregulation of IL-1β and TNF-α expression (Fig. 4b, c).

After ischemia has started, energy depletion causes mitochondrial dysfunction and early generation of ROS and reactive nitrogen species (RNS) [19], the accumulation of which triggers inflammation through the initiation of a chain of harmful cell responses [48]. ROS are molecules derived from small oxygen species, including the superoxide anion radical (O_2_·−), hydroxyl radical (OH·), and certain non-radicals, such as hydrogen peroxide (H_2_O_2_) and the oxygen singlet (^1^O_2_) [27]. Among these oxidants, the superoxide anion is the most destructive because it can cause cytotoxicity in combination with NO [49]. In this study, PRex pretreatment effectively inhibited the increase in the ROS and MDA levels caused by MCAO-induced brain injury (Fig. 5a, b). MDA is a lipoperoxidation product that is used as an indicator of oxidative stress [50].

Consequently, PRex pretreatment suppressed infarction in mice brains after MCAO through decreased oxidative stress, owing to reduced iNOS and ROS, and the regulation of the expression of pro-inflammatory factors, such as IL-1β and TNF-α. These results indicate that neuro-protective effects of PRex could be mediated by anti-oxidative and anti-inflammatory mechanisms in MCAO mice model ((Additional file 1: Figure S2).

Based on above results, PR could be regarded as promising agent for the prevention or initial treatment of stroke through the inhibition of inflammatory responses in cerebral infarction. However, further pharmacological studies on the effects of the pretreatment of other extracts of PR on stroke, the components of PR that affect each pathway of ischemia-induced brain damage, and the effects of PR treatment after cerebral damage are required to establish to clinical applications.

## Conclusions

To demonstrate the effects of PRex pretreatment on ischemia-induced brain damage, the infarct volumes in the brain, the neurological deficit scores, and the expression of oxidative stress factors and inflammatory cytokines were observed at 24 h after MCAO-induced brain injury in mice. The results indicated that PRex pretreatment significantly decreased the infarct volume in mice brains after MCAO, but resulted in no significant reduction in the neurological behavioral deficit. PRex pretreatment significantly inhibited the expression of iNOS and the levels of ROS and MDA in mice brains after MCAO. PRex pretreatment significantly suppressed the expression of IL-1β and TNF-α in mice brains after MCAO. In conclusion, PRex pretreatment reduced the infarct volumes in the brains of mice with MCAO-induced brain injury through interference in the inflammatory responses of ischemic brain injury. Our results indicated that PR could be a potential candidate for the prevention or treatment of cerebral stroke.

## Additional file

**Additional file 1: Figure S1.** High performance thin layer chromatography (HPTLC) images of methanol extract of Peucedani Radix (PRex) fingerprinting. **Figure S2.** Schematic view of neuro-protective mechanisms of PRex in MCAO mice model.

**Additional file 2** The Minimum Standards of Reporting Checklist.

## Abbreviations

PR: Peucedani Radix
PRex: methanol extract of Peucedani Radix
MCAO: middle cerebral artery occlusion
DCFH-DA: dichlorofluorescein diacetate
TBARS: thiobarbituric acid-reactive substances
ELISA: enzyme-linked immunosorbent assay
iNOS: inducible nitric oxide synthase
ROS: reactive oxygen species
MDA: malondialdehyde
IL: interleukin
TNF-α: tumor necrosis factor alpha
