# Supplementary material for: Effects of pretreatment with methanol extract of Peucedani Radix on transient ischemic brain injury in mice
Source: Chin Med. 2017 Oct 24;12:30. doi: 10.1186/s13020-017-0151-z (PMC5655947; doi:10.1186/s13020-017-0151-z)
Supplement: Supplementary file 1 — Additional file 1: Figure S1. High performance thin layer chromatography (HPTLC) images of methanol extract of Peucedani Radix (PRex) fingerprinting. Figure S2. Schematic view of neuro-protective mechanisms of PRex in MCAO mice model. [file 13020_2017_151_MOESM1_ESM.docx]

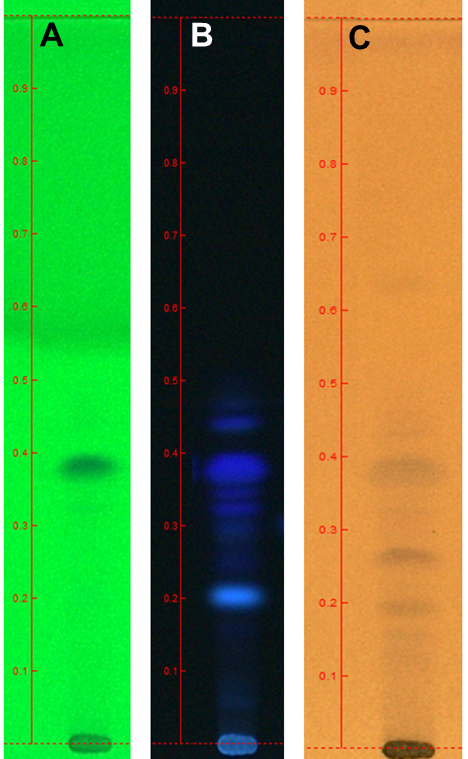


**Figure S1. High performance thin layer chromatography (HPTLC) images of PRex fingerprinting**. HPTLC plate (silica gel F254; mobile phase, chloroform:methanol/30:1) and visualizer (Camag, Swiss) were used in acquiring the images. A, 254 nm UV; B, 366 nm UV; C, white light; D, p-anisaldehyde sprayed followed by white light detected.


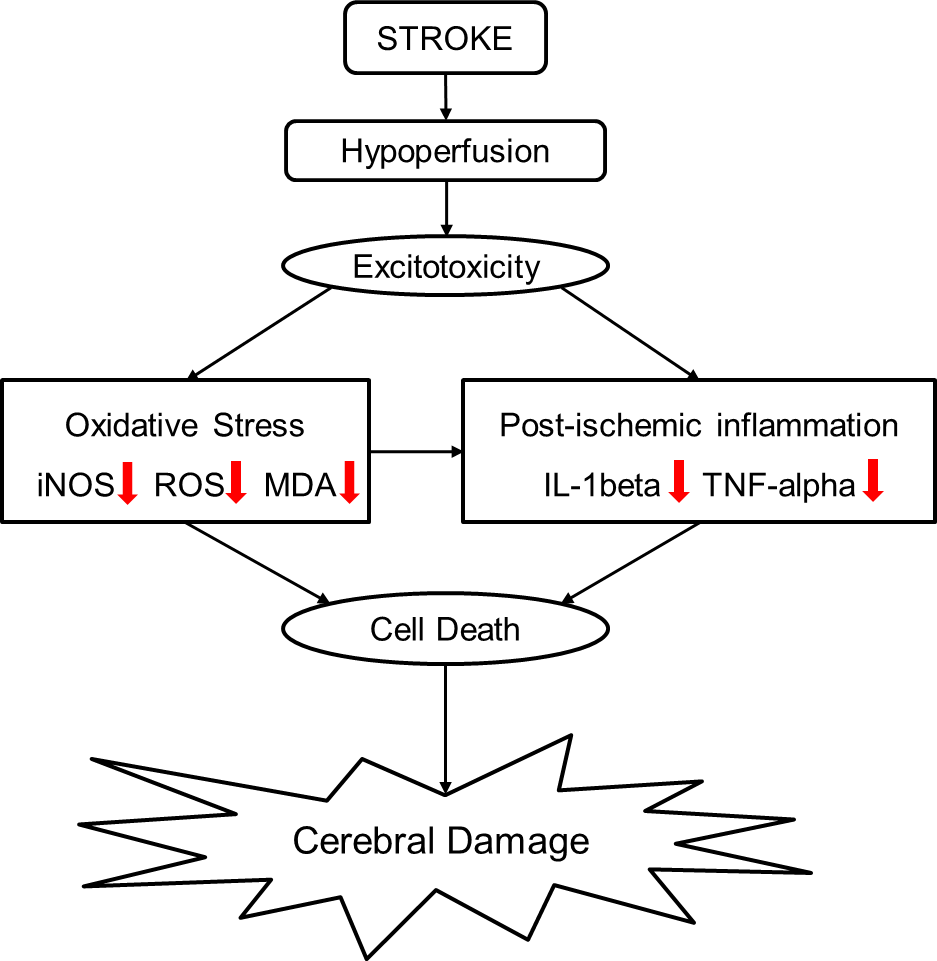


**Figure S2. Schematic view of anti-oxidant and anti-inflammatory mechanism of PRex in MCAO mice model.** Red arrows represent expected effects of PRex on brain injury induced by MCAO.
